# Recent advances in echocardiography for valvular heart disease

**DOI:** 10.12688/f1000research.6446.1

**Published:** 2015-09-28

**Authors:** Rebecca Hahn

**Affiliations:** 1 Columbia University Medical Center/New York Presbyterian Hospital, New York, NY, USA

**Keywords:** echocardiography, valvular heart disease, three-dimensional echocardiography, strain imaging, intracardiac echocardiography, fusion imaging

## Abstract

Echocardiography is the imaging modality of choice for the assessment of patients with valvular heart disease. Echocardiographic advancements may have particular impact on the assessment and management of patients with valvular heart disease. This review will summarize the current literature on advancements, such as three-dimensional echocardiography, strain imaging, intracardiac echocardiography, and fusion imaging, in this patient population.

## Introduction

The American Heart Association/American College of Cardiology Guidelines for the Management of Patients with Valvular Heart Disease ^1^ state that echocardiography (transthoracic [TTE] or transesophageal [TEE]) is the imaging modality of choice for the assessment of patients with valvular heart disease. Numerous less invasive therapies, such as percutaneous or transcatheter interventions, have recently been introduced for the treatment of structural heart disease. Many of these procedures require extensive multi-modality imaging guidance and have increased interest in advancements in echocardiography. Recent advancements in echocardiography are of particular relevance to valvular disease. This review will discuss the application of these new technologies to diagnose and manage various types of valvular heart diseases.

## Three-dimensional echocardiography

The echocardiographic advancement that has had the most impact on the diagnosis of valvular heart disease is real time three-dimensional (RT3D) echocardiography. The advantages of three-dimensional (3D) imaging over two-dimensional (2D) imaging has been well described in the most recent societal guidelines: “Recommendations for cardiac chamber quantification by echocardiography in adults” update ^2^ and “Recommendations for image acquisition and display using 3D echocardiography” ^3^. These guidelines review the significant data supporting the improved accuracy and reproducibility of 3D imaging for ventricular volumes and mass, as well as valvular morphology and function. Initially introduced in the year 2000, the continued improvement of 3D technology has led to its widespread availability and its growing utility, particularly for valvular heart disease ^4^.

### Valve morphology

RT3D TEE has significantly changed the assessment of valvular pathology and has revolutionized patient selection not only for surgical repair but also for newer transcatheter procedures, discussed in a subsequent section.

RT3D TEE is not only more accurate than 2D techniques in identifying specific mitral valve pathology in the setting of complex disease but the diagnosis can be made more rapidly, which is of particular use in the intraoperative evaluation of patients undergoing mitral valve repair ^5– 9^. RT3D echo improves the accuracy and reproducibility of planimetry measurements of mitral valve area in the setting of rheumatic disease by ensuring on-axis imaging of the short-axis view ^10– 13^. This technology has also been integral to our understanding of the dynamic nature of the mitral valve complex in normal patients, as well as in primary and secondary mitral valve disease ^14– 18^.

Recent RT3D TEE studies have shown a coupling of mitral and aortic valve dynamic anatomy. Mitral valve diseases may affect normal mitral-aortic coupling and aortic valve function; different patterns of abnormal mitral-aortic coupling are associated with different Carpentier types of mitral regurgitation ^19^. Conversely, changes in aortic morphology may affect mitral valve function, particularly in the setting of aortic stenosis and calcification of the aortic-mitral fibrous continuity ^20^. RT3D TEE has shown changes in mitral valve morphology following surgical aortic valve replacement ^21^ as well as transcatheter aortic valve replacement (TAVR) ^22^. Notably, a decrease of tenting area predicted those patients whose mitral regurgitation improved following TAVR.

Aortic valve morphology as well as aortic root measurements are more accurate and reproducible with RT3D imaging. Inter-commissural distance and free leaflet edge lengths can be measured by 3D echocardiography and are used to choose the tube graft size in valve-sparing root operations ^23^. Larger left ventricular outflow tract areas and calculated aortic valve dimensions and areas are obtained by RT3D TEE ^24^. Planimetry of the aortic valve and left ventricular outflow tract area by RT3D has been shown to be accurate and reproducible ^25– 27^ and may influence surgical decision-making in the setting of moderate-to-severe aortic stenosis ^28^. With accurate measurement of the left ventricular outflow tract, geometric assumptions used in the continuity equation are avoided, resulting in more precise estimations of aortic valve areas using 3D echocardiography over traditional 2D methods.

Prosthetic valve function can also be accurately assessed using RT3D TEE. With transcatheter solutions to bioprosthetic valve failure ^29– 31^ and paravalvular regurgitation ^32– 34^ RT3D TEE has become an important tool for intra-procedural guidance during percutaneous interventions. TEE can depict not only the relevant cardiac landmarks adjacent to the sites of paravalvular leaks but also wires, delivery catheters, and closure devices ^35^. RT3D TEE imaging results in a more accurate localization of paravalvular defects and an estimation of the size of the defect that correlated better with surgical findings when compared with 2D TEE ^36^.

### Quantification of valvular function

Three-dimensional color Doppler may overcome the limitations of 2D and standard Doppler measurements for quantifying regurgitation ^3, 37, 38^. Studies have shown the feasibility of measuring the 3D vena contracta (narrowest portion of the regurgitant jet) on RT3D echocardiography to assess the severity of regurgitation for native regurgitant valve disease ^37, 39– 41^, as well as following surgical ^42^ or transcatheter interventions ^43^.

Calculation of regurgitant volume in native valvular disease using the proximal isovelocity surface area (PISA) method ^44^ has known technical limitations, primarily the geometric assumptions of PISA shape required to calculate effective regurgitant orifice area. Multiple studies have validated the use of single-beat RT3D echocardiographic color Doppler imaging allowing the direct measurement of PISA without geometric assumptions for aortic, mitral, and tricuspid regurgitation assessment ^45– 48^.

Newer methods of determining relative flows within the heart make use of the velocity and direction of flow information which can be derived from color Doppler. Off-line software has been developed which uses 2D color Doppler images to determine the velocity, flow rate, and flow volume in any given region of the heart ^49^. Extension of this technology to 3D color Doppler volume sets is now possible and allows rapid, accurate, and reproducible quantitation of relative stroke volumes ^50, 51^. Thavendiranathan *et al.* ^51^ used the velocity information encoded in the volume color Doppler data, targeting the appropriate region of interest by using the simultaneous 3D imaging of the mitral annulus and left ventricular outflow tract. Color Doppler velocity is multiplied by a known area of this cross-section (a voxel area), and the resulting spatially averaged flow rates are used to generate flow-time curves that resemble those obtained by magnetic resonance imaging. The temporal integration of the flow-time curve generates the stroke volume. There was excellent correlation between the automated measured mitral inflow and aortic stroke volumes, and magnetic resonance imaging stroke volume (r = 0.91, 95% confidence interval [CI], 0.83–0.95, and r = 0.93, 95% CI, 0.87–0.96, respectively, *P*<0.001) and very low interobserver variability. Automation of the measurement process allowed calculations of mitral inflow and aortic stroke volumes to be performed very rapidly. This methodology will likely become the standard for measurement of regurgitant volumes in the future.

### Structural heart disease interventions

TAVR has become an acceptable alternative treatment for high-risk or inoperable patients with severe symptomatic aortic stenosis ^52– 55^. Three-dimensional echocardiography has been shown to improve sizing of the transcatheter valve ^56– 58^. RT3D TEE is comparable to computed tomography for annular assessment and prediction of paravalvular regurgitation due to oversizing ^59, 60^, as well as measurement of coronary artery height ^61^. RT3D TEE has been shown to provide superior spatial visualization and anatomic orientation, optimizing procedural performance, and RT3D TTE can be used to assess the severity of paravalvular regurgitation following TAVR ^62^. Further study of this technique for quantifying regurgitant severity is warranted in addition to a unified scheme for grading paravalvular regurgitation following TAVR ^63^. Newer devices, with features such as external skirts or the ability to reposition, may reduce the incidence of post-TAVR complication.

**Figure 1.**
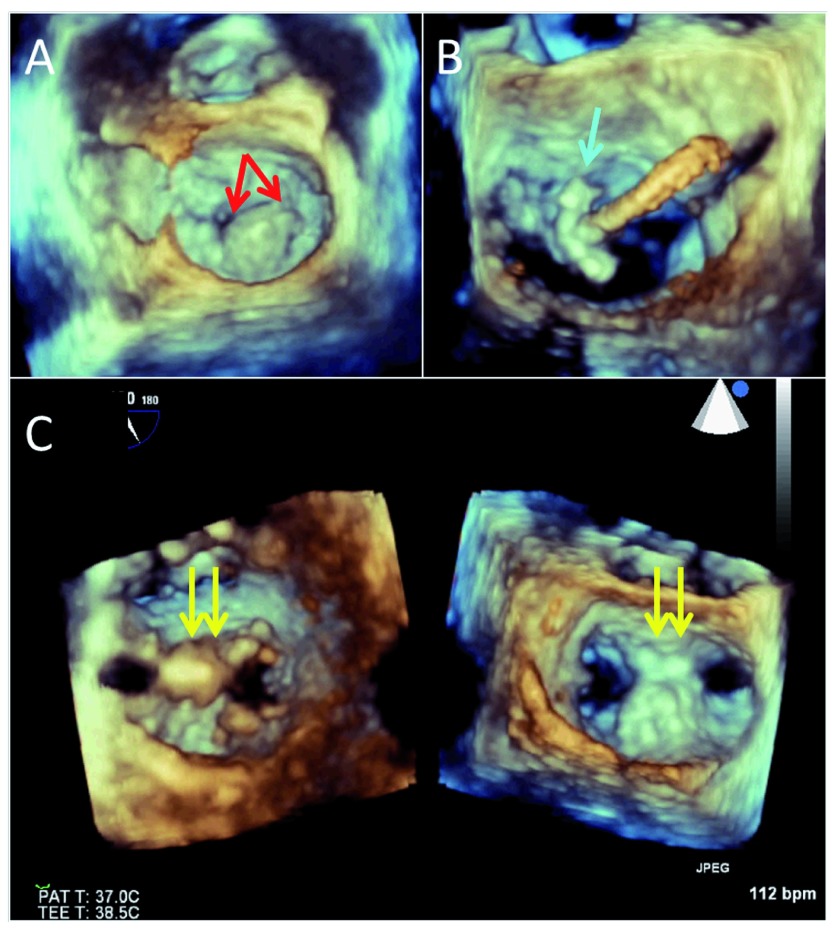
Three-dimensional echocardiography during a transcatheter mitral repair procedure. Panel A shows the baseline mitral valve morphology with a very large prolapsing and partially flail P2 (middle) scallop (red arrows). Panel B shows positioning of the MitraClip device (blue arrow). Panel C is a dual plane three-dimensional image (ventricular and atrial views) of the final, 2-clip (yellow arrows) resulting double orifice. There was trivial residual mitral regurgitation.

Three-dimensional TEE may also improve procedural success and shorten procedure time for the MitraClip™ device (Abbott Vascular Structural Heart, Menlo Park, CA) ( Figure 1) ^64– 66^. Altiok *et al.* ^65^ performed a structured analysis to compare information and guidance capability provided by RT3D TEE compared to 2D TEE and found 3D TEE advantageous in 9 of 11 steps of the percutaneous mitral repair procedure, including optimizing trans-septal puncture site, guidance of the clip delivery system, precise positioning of the clip delivery system simultaneously in anterior-posterior and lateral-medial direction, valvular regurgitation jet position, adjustment and visualization of the clip position relative to the valvular orifice, and assessment of remaining regurgitant jets ^65^. Following MitraClip, assessment of residual regurgitation could also be assessed by 3D color Doppler ^43^. A >50% reduction in regurgitant volume using the product of vena contracta areas defined by direct planimetry of RT3D color Doppler and velocity time integral using continuous-wave Doppler was associated with greater left atrial and ventricular remodeling.

## Strain imaging

Recent American Society of Echocardiography Chamber Quantification guidelines strongly recommend routine assessment of ventricular systolic function by quantification of ventricular volumes and calculation of ejection fraction (EF) ^2^. Cardiac mechanics, however, can now be assessed with the use of both tissue Doppler and speckle tracking for the measurement of myocardial displacement ^67^. The measurement of myocardial deformation or “strain” is the fractional change in the length of a myocardial segment (expressed as a percentage of the baseline length). Strain rate is the rate of change in strain. The deformation of the myocardium is directional: lengthening would be represented by positive strain, and shortening by negative strain. Systolic strain can be measured along the anatomic coordinates of the cardiac chambers: longitudinal (negative strain), radial (positive strain), and circumferential (negative strain). The strengths and weaknesses of strain measurement have been well described ^67^; however, the recent standardization of strain Digital Imaging and Communications in Medicine (DICOM) format will reduce inter-vendor variability which, along with improved software analysis and automation packages, will likely increase the clinical acceptability and use of this powerful technique.

### Aortic valve disease

**Figure 2.**
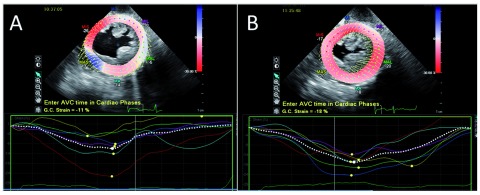
Strain imaging during transcatheter aortic valve replacement (TAVR). Panel A shows a global circumferential strain (GCS) of -11% prior to TAVR. Panel B shows a GCS of -18% following TAVR. This represents an improvement (greater shortening) in ventricular mechanics.

Numerous studies have shown the utility of strain imaging for assessing left ventricular function in aortic valve disease. In the presence of normal EF, increasing severity of aortic stenosis was associated with reduced global longitudinal strain (GLS) ^68, 69^. Subclinical improvement in global and regional systolic function by tissue Doppler and speckle strain also occurs following TAVR ( Figure 2) ^70– 72^. In low flow, low gradient, severe aortic stenosis with normal EF, strain parameters improved following TAVR, even in the absence of significant change in EF ^73^. Regional strain abnormalities in patients with severe aortic stenosis may be able to further sub-stratify patients with concomitant infiltrative diseases, such as amyloid as well as coronary disease. In patients with cardiac amyloid, relative apical sparing (with preserved apical longitudinal strain) was sensitive (93%) and specific (82%) in differentiating amyloid from controls, some of whom had severe aortic stenosis. In patients with moderate or severe aortic stenosis and concomitant coronary disease, on the other hand, worse apical and mid longitudinal strain parameters were predictive of significant coronary artery stenosis ^74^.

Because mortality is significantly associated with symptom development ^75^, strain has been postulated as a possible early marker of ventricular dysfunction in asymptomatic patients with severe aortic stenosis and thus may be a useful tool in determining the timing of intervention in this population. In fact, Carasso *et al.* ^76^ showed that longitudinal strain was low in asymptomatic patients with severe aortic stenosis with supernormal apical circumferential strain and rotation. In symptomatic patients, however, longitudinal strain was significantly lower with no compensatory circumferential myocardial mechanics. Other investigators suggest that, after adjusting for aortic stenosis severity and EF, only basal longitudinal strain (and not GLS) was an independent predictor of symptomatic status ^77^. In fact, following TAVR, the improvement in GLS may be a result of basal and mid segment improvement only ^78^.

Strain imaging may be particularly useful in predicting outcomes in patients with severe aortic stenosis. In patients with low flow, low gradient, aortic stenosis with normal EF, a recent study showed both stroke volume index (≤35 ml/m ^2^) and GLS (>-15%) are independently associated with worse survival ^79^. In patients with low flow, low gradient, aortic stenosis with reduced EF, GLS is independently associated with mortality and dobutamine stress GLS may provide incremental prognostic value beyond GLS measured at rest ^80^. Three-dimensional GLS may be a better predictor of outcome compared to 2D strain ^81^. Finally, Kusunose *et al*. ^82^ studied 395 patients with moderate-severe aortic stenosis (aortic valve area <1.3 cm ^2^) and found that GLS was an independent predictor of mortality in this population. A GLS >-12% was associated with the lowest survival ^82^.

Deformation characteristics have also been studied in patients with aortic regurgitation ^83– 87^. In a prospective study of young patients (<18 years old) with aortic regurgitation, the only significant predictor of progression of disease on multi-variable analysis was GLS ( *P*=0.04, cut-off value of >-19.5%, sensitivity of 77.8%, specificity of 94.1%, and area under the curve of 0.89) ^83^. Prospective studies of adult patients have also shown that strain parameters by speckle-tracking could detect early myocardial systolic and diastolic dysfunction, and lower strain values were associated with disease progression in medically managed patients, or impaired outcomes in surgically treated patients ^85^. A systolic radial strain rate of <1.82/sec was a good predictor of postoperative left ventricular dysfunction ^86^. Finally, in a prospective study, 60 patients with chronic aortic regurgitation were followed for 64 months and global longitudinal strain (four-chamber view only) was an independent predictor of mortality (hazard ratio 1.313, 95% CI 1.010-1.706, *P*=0.042) ^87^.

### Mitral valve disease

Chronic mitral regurgitation is associated with complex left ventricular adaptive remodeling, eccentric hypertrophy, and, eventually, reduced EF. Current guidelines recommend intervening on severe, asymptomatic mitral regurgitation in the setting of reduced EF because of a high incidence of persistent or worsening dysfunction ^88^. In chronic severe degenerative mitral regurgitation, numerous studies have shown that a reduced baseline GLS signifies a maladaptive preload-related change that is associated with a reduction in left ventricular EF immediately after mitral valve repair ^89– 91^. A GLS cutoff of >-19.9% was a strong independent predictor of long-term left ventricular dysfunction and may become an appropriate indication for intervention in the setting of normal EF ^90^.

## Intra-cardiac echocardiography

**Figure 3.**
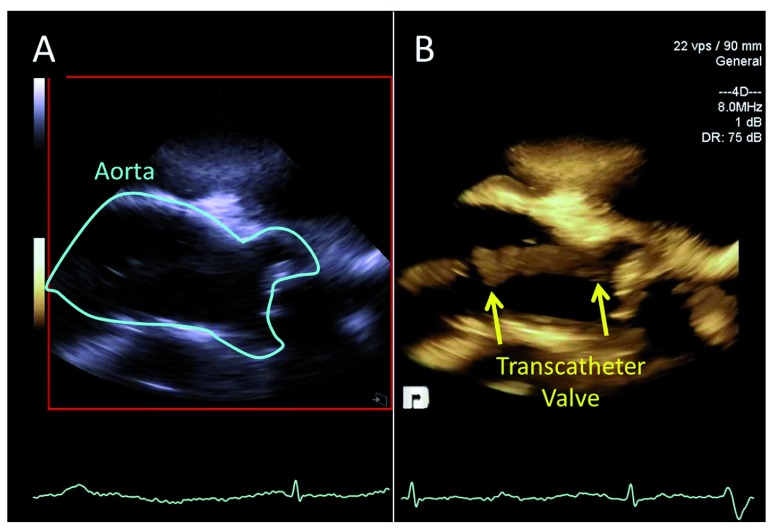
Intra-cardiac echocardiography (ICE) during transcatheter aortic valve replacement (TAVR). Panel A is the two-dimensional ICE image with panel B showing the simultaneous three-dimensional volume during positioning of the transcatheter aortic valve (yellow arrows).

Although TEE imaging is well established and provides exceptional images, particularly for intra-procedural guidance, it most commonly requires general anesthesia and may be associated with intermittent obstruction of fluoroscopic viewing ^92^. With the current move toward conscious sedation for structural heart disease interventions, intra-cardiac echocardiography (ICE) may be an acceptable alternative in some patients with no other adequate intra-procedural imaging options. Evidence that ICE guidance can improve safety and outcome of interventional procedures is still lacking; however, ICE imaging for paravalvular leak closure has been reported to be feasible and advantageous ^32, 93^. A reduction in contrast use has also been reported with 2D ICE when used in TAVR ( Figure 3) ^94^. The recently introduced AcuNav ^®^ V catheter (Siemens Inc. Mountain View, USA) represents the only commercially available RT3D ICE system. The 10F catheter carries a matrix transducer providing a 22° × 90° real-time volume image. This small volume represents the main limitation, particularly in near field applications, such as structural heart disease interventions.

## Fusion imaging

Combining images from two or more different imaging techniques, or fusion imaging, has been accomplished most recently with real-time echocardiography and fluoroscopy ^95– 97^. This technology, which co-registers the TEE probe position with the intervention table and the angulation of the fluoroscopy C-arm, allows for relatively accurate placement of the TEE image onto the fluoroscopic image. This integration eliminates the need for two different image display monitors and the mental integration of two very different imaging datasets by the operator of structural heart disease interventions.

**Figure 4.**
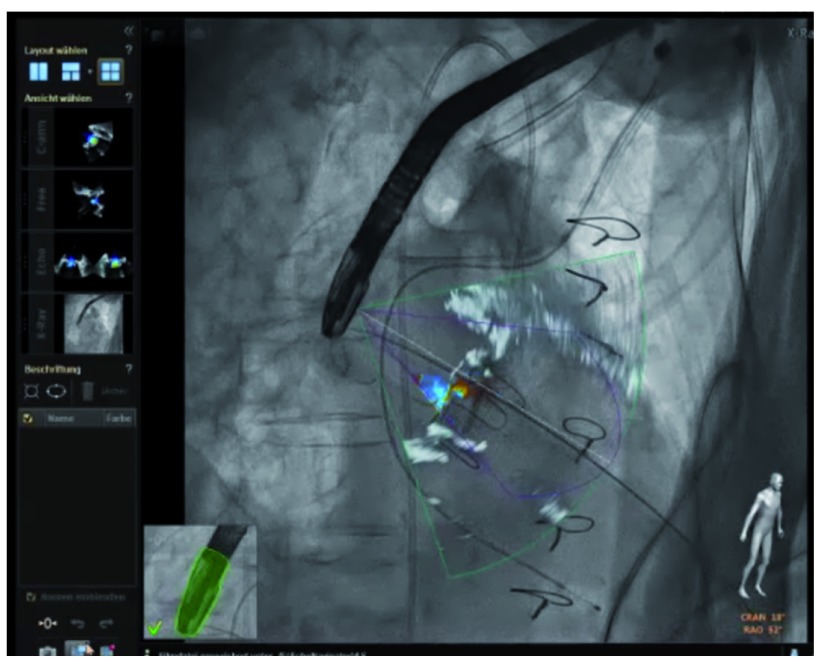
Hybrid/fusion imaging during paravalvular regurgitation closure. After coregistration of the transesophageal echocardiographic probe with the fluoroscopic image, the two images can be fused to allow a more comprehensive understanding of anatomy. Localizing the regurgitant orifice on transesophageal echo imaging can then be translated to the corresponding position on the fluoroscopic image.

The ability to define targets on echocardiographic images (whether 2D or 3D), and co-register these targets onto the fluoroscopic images, should improve guidance of structural heart disease interventions ( Figure 4). This technology has been shown to be safe and feasible for the transcatheter mitral repair procedure with the MitraClip™ device (Abbott Vascular Structural Heart, Menlo Park, CA) and shows a trend towards reduction of fluoroscopy and procedure time ^98^.

## Conclusion

Echocardiography is the primary imaging modality for the diagnosis and management of patients with valvular heart disease. Improvement in surgical outcomes and advances in interventional techniques require further refinements in echocardiographic imaging. Three-dimensional echocardiography, strain imaging, intracardiac echocardiography, and fusion imaging have significant application in advancing our understanding of pathophysiology and anatomy, as well as the diagnosis and management of patients with valvular heart disease.

## Abbreviations

2D, two-dimensional; 3D, three-dimensional; confidence interval, CI; EF, ejection fraction; GLS, global longitudinal strain; ICE, intra-cardiac echocardiography; PISA, proximal isovelocity surface area; RT3D, real time three-dimensional; TAVR, transcatheter aortic valve replacement; TEE, transesophageal echocardiography; TTE, transthoracic echocardiography.

## References

[1] Nishimura RA Otto C: 2014 ACC/AHA valve guidelines: earlier intervention for chronic mitral regurgitation. *Heart.* 2014;100(12):905–7. 10.1136/heartjnl-2014-305834 24688115

[2] Lang RM Badano LP Mor-Avi V: Recommendations for cardiac chamber quantification by echocardiography in adults: an update from the American Society of Echocardiography and the European Association of Cardiovascular Imaging. *J Am Soc Echocardiogr.* 2015;28(1):1–39.e14. 10.1016/j.echo.2014.10.003 25559473

[3] Lang RM Badano LP Tsang W: EAE/ASE recommendations for image acquisition and display using three-dimensional echocardiography. *J Am Soc Echocardiogr.* 2012;25(1):3–46. 10.1016/j.echo.2011.11.010 22183020

[4] Lang RM Badano LP Tsang W: EAE/ASE recommendations for image acquisition and display using three-dimensional echocardiography. *Eur Heart J Cardiovasc Imaging.* 2012;13(1):1–46. 10.1093/ehjci/jer316 22275509

[5] Grewal J Mankad S Freeman WK: Real-time three-dimensional transesophageal echocardiography in the intraoperative assessment of mitral valve disease. *J Am Soc Echocardiogr.* 2009;22(1):34–41. 10.1016/j.echo.2008.11.008 19131000

[6] Pepi M Tamborini G Maltagliati A: Head-to-head comparison of two- and three-dimensional transthoracic and transesophageal echocardiography in the localization of mitral valve prolapse. *J Am Coll Cardiol.* 2006;48(12):2524–30. 10.1016/j.jacc.2006.02.079 17174193

[7] Ben Zekry S Nagueh SF Little SH: Comparative accuracy of two- and three-dimensional transthoracic and transesophageal echocardiography in identifying mitral valve pathology in patients undergoing mitral valve repair: initial observations. *J Am Soc Echocardiogr.* 2011;24(10):1079–85. 10.1016/j.echo.2011.06.011 21803543

[8] Hien MD Rauch H Lichtenberg A: Real-time three-dimensional transesophageal echocardiography: improvements in intraoperative mitral valve imaging. *Anesth Analg.* 2013;116(2):287–95. 10.1213/ANE.0b013e318262e154 22798535

[9] Levack MM Jassar AS Shang EK: Three-dimensional echocardiographic analysis of mitral annular dynamics: implication for annuloplasty selection. *Circulation.* 2012;126(11 Suppl 1):S183–8. 10.1161/CIRCULATIONAHA.111.084483 22965981 PMC3458517

[10] Zamorano J Cordeiro P Sugeng L: Real-time three-dimensional echocardiography for rheumatic mitral valve stenosis evaluation: an accurate and novel approach. *J Am Coll Cardiol.* 2004;43(11):2091–6. 10.1016/j.jacc.2004.01.046 15172418

[11] Schlosshan D Aggarwal G Mathur G: Real-time 3D transesophageal echocardiography for the evaluation of rheumatic mitral stenosis. *JACC Cardiovasc Imaging.* 2011;4(6):580–8. 10.1016/j.jcmg.2010.12.009 21679891

[12] Min SY Song JM Kim YJ: Discrepancy between mitral valve areas measured by two-dimensional planimetry and three-dimensional transoesophageal echocardiography in patients with mitral stenosis. *Heart.* 2013;99(4):253–8. 10.1136/heartjnl-2012-302742 23125249

[13] Wunderlich NC Beigel R Siegel RJ: Management of mitral stenosis using 2D and 3D echo-Doppler imaging. *JACC Cardiovasc Imaging.* 2013;6(11):1191–205. 10.1016/j.jcmg.2013.07.008 24229772

[14] Levine RA Handschumacher MD Sanfilippo AJ: Three-dimensional echocardiographic reconstruction of the mitral valve, with implications for the diagnosis of mitral valve prolapse. *Circulation.* 1989;80(3):589–98. 10.1161/01.CIR.80.3.589 2766511

[15] Topilsky Y Vaturi O Watanabe N: Real-time 3-dimensional dynamics of functional mitral regurgitation: a prospective quantitative and mechanistic study. *J Am Heart Assoc.* 2013;2(3):e000039. 10.1161/JAHA.113.000039 23727698 PMC3698758

[16] Grewal J Suri R Mankad S: Mitral annular dynamics in myxomatous valve disease: new insights with real-time 3-dimensional echocardiography. *Circulation.* 2010;121(12):1423–31. 10.1161/CIRCULATIONAHA.109.901181 20231533

[17] Watanabe N Ogasawara Y Yamaura Y: Mitral annulus flattens in ischemic mitral regurgitation: geometric differences between inferior and anterior myocardial infarction: a real-time 3-dimensional echocardiographic study. *Circulation.* 2005;112(9 Suppl):I458–62. 16159863 10.1161/CIRCULATIONAHA.104.524595

[18] Zeng X Nunes MCP Dent J: Asymmetric versus symmetric tethering patterns in ischemic mitral regurgitation: geometric differences from three-dimensional transesophageal echocardiography. *J Am Soc Echocardiogr.* 2014;27(4):367–75. 10.1016/j.echo.2014.01.006 24513242

[19] Looi JL Lee AP Fang F: Abnormal mitral-aortic intervalvular coupling in mitral valve diseases: a study using real-time three-dimensional transesophageal echocardiography. *Clin Res Cardiol.* 2015. 10.1007/s00392-015-0851-2 25855394

[20] Tsang W Meineri M Hahn RT: A three-dimensional echocardiographic study on aortic-mitral coupling in transcatheter aortic valve replacement. *Eur Heart J Cardiovasc Imaging.* 2013;14(10):950–6. 10.1093/ehjci/jet058 23720379

[21] Mahmood F Warraich HJ Gorman JH 3rd: Changes in mitral annular geometry after aortic valve replacement: a three-dimensional transesophageal echocardiographic study. *J Heart Valve Dis.* 2012;21(6):696–701. 23409347 PMC4104570

[22] Shibayama K Harada K Berdejo J: Effect of transcatheter aortic valve replacement on the mitral valve apparatus and mitral regurgitation: real-time three-dimensional transesophageal echocardiography study. *Circ Cardiovasc Imaging.* 2014;7(2):344–51. 10.1161/CIRCIMAGING.113.000942 24474596

[23] Otani K Takeuchi M Kaku K: Assessment of the aortic root using real-time 3D transesophageal echocardiography. *Circ J.* 2010;74(12):2649–57. 10.1253/circj.CJ-10-0540 21084759

[24] Saitoh T Shiota M Izumo M: Comparison of left ventricular outflow geometry and aortic valve area in patients with aortic stenosis by 2-dimensional versus 3-dimensional echocardiography. *Am J Cardiol.* 2012;109(11):1626–31. 10.1016/j.amjcard.2012.01.391 22440128

[25] Goland S Trento A Iida K: Assessment of aortic stenosis by three-dimensional echocardiography: an accurate and novel approach. *Heart.* 2007;93(7):801–7. 10.1136/hrt.2006.110726 17488766 PMC1994466

[26] Doddamani S Bello R Friedman MA: Demonstration of left ventricular outflow tract eccentricity by real time 3D echocardiography: implications for the determination of aortic valve area. *Echocardiography.* 2007;24(8):860–6. 10.1111/j.1540-8175.2007.00479.x 17767537

[27] Shahgaldi K Manouras A Brodin LÅ: Direct measurement of left ventricular outflow tract area using three-dimensional echocardiography in biplane mode improves accuracy of stroke volume assessment. *Echocardiography.* 2010;27(9):1078–85. 10.1111/j.1540-8175.2010.01197.x 20546012

[28] Jainandunsing JS Mahmood F Matyal R: Impact of three-dimensional echocardiography on classification of the severity of aortic stenosis. *Ann Thorac Surg.* 2013;96(4):1343–8. 10.1016/j.athoracsur.2013.05.018 23891412

[29] Dvir D Barbanti M Tan J: Transcatheter aortic valve-in-valve implantation for patients with degenerative surgical bioprosthetic valves. *Curr Probl Cardiol.* 2014;39(1):7–27. 10.1016/j.cpcardiol.2013.10.001 24331437

[30] Cheung A Webb JG Barbanti M: 5-year experience with transcatheter transapical mitral valve-in-valve implantation for bioprosthetic valve dysfunction. *J Am Coll Cardiol.* 2013;61(17):1759–66. 10.1016/j.jacc.2013.01.058 23500301

[31] Cullen MW Cabalka AK Alli OO: Transvenous, antegrade Melody valve-in-valve implantation for bioprosthetic mitral and tricuspid valve dysfunction: a case series in children and adults. *JACC Cardiovasc Interv.* 2013;6(6):598–605. 10.1016/j.jcin.2013.02.010 23683739

[32] Rihal CS Sorajja P Booker JD: Principles of percutaneous paravalvular leak closure. *JACC Cardiovasc Interv.* 2012;5(2):121–30. 10.1016/j.jcin.2011.11.007 22361595

[33] Ruiz CE Jelnin V Kronzon I: Clinical outcomes in patients undergoing percutaneous closure of periprosthetic paravalvular leaks. *J Am Coll Cardiol.* 2011;58(21):2210–7. 10.1016/j.jacc.2011.03.074 22078427

[34] Nietlispach F Johnson M Moss RR: Transcatheter closure of paravalvular defects using a purpose-specific occluder. *JACC Cardiovasc Interv.* 2010;3(7):759–65. 10.1016/j.jcin.2010.04.013 20650438

[35] Marsan NA Tops LF Nihoyannopoulos P: Real-time three dimensional echocardiography: current and future clinical applications. *Heart.* 2009;95(22):1881–90. 10.1136/hrt.2008.151613 19875375

[36] Singh P Manda J Hsiung MC: Live/real time three-dimensional transesophageal echocardiographic evaluation of mitral and aortic valve prosthetic paravalvular regurgitation. *Echocardiography.* 2009;26(8):980–7. 10.1111/j.1540-8175.2009.01022.x 19968687

[37] Perez de Isla L Zamorano J Fernandez-Golfin C: 3D color-Doppler echocardiography and chronic aortic regurgitation: a novel approach for severity assessment. *Int J Cardiol.* 2013;166(3):640–5. 10.1016/j.ijcard.2011.11.094 22192301

[38] Gonçalves A Almeria C Marcos-Alberca P: Three-dimensional echocardiography in paravalvular aortic regurgitation assessment after transcatheter aortic valve implantation. *J Am Soc Echocardiogr.* 2012;25(1):47–55. 10.1016/j.echo.2011.08.019 21962448

[39] Mori Y Shiota T Jones M: Three-dimensional reconstruction of the color Doppler-imaged vena contracta for quantifying aortic regurgitation: studies in a chronic animal model. *Circulation.* 1999;99(12):1611–7. 10.1161/01.CIR.99.12.1611 10096939

[40] Fang L Hsiung MC Miller AP: Assessment of aortic regurgitation by live three-dimensional transthoracic echocardiographic measurements of vena contracta area: usefulness and validation. *Echocardiography.* 2005;22(9):775–81. 10.1111/j.1540-8175.2005.00171.x 16194172

[41] Chen TE Kwon SH Enriquez-Sarano M: Three-dimensional color Doppler echocardiographic quantification of tricuspid regurgitation orifice area: comparison with conventional two-dimensional measures. *J Am Soc Echocardiogr.* 2013;26(10):1143–52. 10.1016/j.echo.2013.07.020 23993694

[42] Franco E Almería C de Agustín JA: Three-dimensional color Doppler transesophageal echocardiography for mitral paravalvular leak quantification and evaluation of percutaneous closure success. *J Am Soc Echocardiogr.* 2014;27(11):1153–63. 10.1016/j.echo.2014.08.019 25260434

[43] Altiok E Hamada S Brehmer K: Analysis of procedural effects of percutaneous edge-to-edge mitral valve repair by 2D and 3D echocardiography. *Circ Cardiovasc Imaging.* 2012;5(6):748–55. 10.1161/CIRCIMAGING.112.974691 23001897

[44] Zoghbi WA Enriquez-Sarano M Foster E: Recommendations for evaluation of the severity of native valvular regurgitation with two-dimensional and Doppler echocardiography. *J Am Soc Echocardiogr.* 2003;16(7):777–802. 10.1016/S0894-7317(03)00335-3 12835667

[45] Pirat B Little SH Igo SR: Direct measurement of proximal isovelocity surface area by real-time three-dimensional color Doppler for quantitation of aortic regurgitant volume: an *in vitro* validation. *J Am Soc Echocardiogr.* 2009;22(3):306–13. 10.1016/j.echo.2008.11.031 19168322 PMC3348870

[46] Little SH Igo SR Pirat B: *In vitro* validation of real-time three-dimensional color Doppler echocardiography for direct measurement of proximal isovelocity surface area in mitral regurgitation. *Am J Cardiol.* 2007;99(10):1440–7. 10.1016/j.amjcard.2006.12.079 17493476 PMC3348701

[47] de Agustín JA Marcos-Alberca P Fernandez-Golfin C: Direct measurement of proximal isovelocity surface area by single-beat three-dimensional color Doppler echocardiography in mitral regurgitation: a validation study. *J Am Soc Echocardiogr.* 2012;25(8):815–23. 10.1016/j.echo.2012.05.021 22739217

[48] de Agustín JA Viliani D Vieira C: Proximal isovelocity surface area by single-beat three-dimensional color Doppler echocardiography applied for tricuspid regurgitation quantification. *J Am Soc Echocardiogr.* 2013;26(9):1063–72. 10.1016/j.echo.2013.06.006 23860094

[49] Li C Zhang J Li X: Quantification of chronic aortic regurgitation by vector flow mapping: a novel echocardiographic method. *Eur J Echocardiogr.* 2010;11(2):119–24. 10.1093/ejechocard/jep175 19933519

[50] Little SH Igo SR McCulloch M: Three-dimensional ultrasound imaging model of mitral valve regurgitation: design and evaluation. *Ultrasound Med Biol.* 2008;34(4):647–54. 10.1016/j.ultrasmedbio.2007.08.009 18255217 PMC3348787

[51] Thavendiranathan P Liu S Datta S: Automated quantification of mitral inflow and aortic outflow stroke volumes by three-dimensional real-time volume color-flow Doppler transthoracic echocardiography: comparison with pulsed-wave Doppler and cardiac magnetic resonance imaging. *J Am Soc Echocardiogr.* 2012;25(1):56–65. 10.1016/j.echo.2011.10.004 22105057

[52] Leon MB Smith CR Mack M: Transcatheter aortic-valve implantation for aortic stenosis in patients who cannot undergo surgery. *N Engl J Med.* 2010;363(17):1597–607. 10.1056/NEJMoa1008232 20961243

[53] Smith CR Leon MB Mack MJ: Transcatheter versus surgical aortic-valve replacement in high-risk patients. *N Engl J Med.* 2011;364(23):2187–98. 10.1056/NEJMoa1103510 21639811

[54] Popma JJ Adams DH Reardon MJ: Transcatheter aortic valve replacement using a self-expanding bioprosthesis in patients with severe aortic stenosis at extreme risk for surgery. *J Am Coll Cardiol.* 2014;63(19):1972–81. 10.1016/j.jacc.2014.02.556 24657695

[55] Adams DH Popma JJ Reardon MJ: Transcatheter aortic-valve replacement with a self-expanding prosthesis. *N Engl J Med.* 2014;371(10):967–8. 10.1056/NEJMc1408396 25184874

[56] Altiok E Koos R Schröder J: Comparison of two-dimensional and three-dimensional imaging techniques for measurement of aortic annulus diameters before transcatheter aortic valve implantation. *Heart.* 2011;97(19):1578–84. 10.1136/hrt.2011.223974 21700756

[57] Gripari P Ewe SH Fusini L: Intraoperative 2D and 3D transoesophageal echocardiographic predictors of aortic regurgitation after transcatheter aortic valve implantation. *Heart.* 2012;98(16):1229–36. 10.1136/heartjnl-2012-301998 22826560

[58] Hahn RT Khalique O Williams MR: Predicting paravalvular regurgitation following transcatheter valve replacement: utility of a novel method for three-dimensional echocardiographic measurements of the aortic annulus. *J Am Soc Echocardiogr.* 2013;26(9):1043–52. 10.1016/j.echo.2013.07.004 23998695

[59] Jilaihawi H Doctor N Kashif M: Aortic annular sizing for transcatheter aortic valve replacement using cross-sectional 3-dimensional transesophageal echocardiography. *J Am Coll Cardiol.* 2013;61(9):908–16. 10.1016/j.jacc.2012.11.055 23449425

[60] Khalique OK Kodali SK Paradis JM: Aortic annular sizing using a novel 3-dimensional echocardiographic method: use and comparison with cardiac computed tomography. *Circ Cardiovasc Imaging.* 2014;7(1):155–63. 10.1161/CIRCIMAGING.113.001153 24221192

[61] Tamborini G Fusini L Gripari P: Feasibility and accuracy of 3DTEE versus CT for the evaluation of aortic valve annulus to left main ostium distance before transcatheter aortic valve implantation. *JACC Cardiovasc Imaging.* 2012;5(6):579–88. 10.1016/j.jcmg.2012.02.012 22698526

[62] Smith LA Dworakowski R Bhan A: Real-time three-dimensional transesophageal echocardiography adds value to transcatheter aortic valve implantation. *J Am Soc Echocardiogr.* 2013;26(4):359–69. 10.1016/j.echo.2013.01.014 23484436

[63] Pibarot P Hahn RT Weissman NJ: Assessment of paravalvular regurgitation following TAVR: a proposal of unifying grading scheme. *JACC Cardiovasc Imaging.* 2015;8(3):340–60. 10.1016/j.jcmg.2015.01.008 25772838

[64] Biner S Perk G Kar S: Utility of combined two-dimensional and three-dimensional transesophageal imaging for catheter-based mitral valve clip repair of mitral regurgitation. *J Am Soc Echocardiogr.* 2011;24(6):611–7. 10.1016/j.echo.2011.02.005 21435839

[65] Altiok E Becker M Hamada S: Optimized guidance of percutaneous edge-to edge repair of the mitral valve using real-time 3-D transesophageal echocardiography. *Clin Res Cardiol.* 2011;100(8):675–81. 10.1007/s00392-011-0296-1 21369924

[66] Guarracino F Baldassarri R Ferro B: Transesophageal echocardiography during MitraClip® procedure. *Anesth Analg.* 2014;118(6):1188–96. 10.1213/ANE.0000000000000215 24842173

[67] Mor-Avi V Lang RM Badano LP: Current and evolving echocardiographic techniques for the quantitative evaluation of cardiac mechanics: ASE/EAE consensus statement on methodology and indications endorsed by the Japanese Society of Echocardiography. *J Am Soc Echocardiogr.* 2011;24(3):277–313. 10.1016/j.echo.2011.01.015 21338865

[68] Miyazaki S Daimon M Miyazaki T: Global longitudinal strain in relation to the severity of aortic stenosis: a two-dimensional speckle-tracking study. *Echocardiography.* 2011;28(7):703–8. 10.1111/j.1540-8175.2011.01419.x 21564277

[69] Ng ACT Delgado V Bertini M: Alterations in multidirectional myocardial functions in patients with aortic stenosis and preserved ejection fraction: a two-dimensional speckle tracking analysis. *Eur Heart J.* 2011;32(12):1542–50. 10.1093/eurheartj/ehr084 21447510

[70] Sengupta PP Tajik AJ Chandrasekaran K: Twist mechanics of the left ventricle: principles and application. *JACC Cardiovasc Imaging.* 2008;1(3):366–76. 10.1016/j.jcmg.2008.02.006 19356451

[71] Bauer F Eltchaninoff H Tron C: Acute improvement in global and regional left ventricular systolic function after percutaneous heart valve implantation in patients with symptomatic aortic stenosis. *Circulation.* 2004;110(11):1473–6. 10.1161/01.CIR.0000134961.36773.D6 15226213

[72] Delgado M Ruiz M Mesa D: Early improvement of the regional and global ventricle function estimated by two-dimensional speckle tracking echocardiography after percutaneous aortic valve implantation speckle tracking after CoreValve implantation. *Echocardiography.* 2013;30(1):37–44. 10.1111/j.1540-8175.2012.01808.x 22985174

[73] Kamperidis V Joyce E Debonnaire P: Left ventricular functional recovery and remodeling in low-flow low-gradient severe aortic stenosis after transcatheter aortic valve implantation. *J Am Soc Echocardiogr.* 2014;27(8):817–25. 10.1016/j.echo.2014.04.021 24906801

[74] Carstensen HG Larsen LH Hassager C: Association of ischemic heart disease to global and regional longitudinal strain in asymptomatic aortic stenosis. *Int J Cardiovasc Imaging.* 2015;31(3):485–95. 10.1007/s10554-014-0572-z 25404082

[75] Ross J Jr Braunwald E: Aortic stenosis. *Circulation.* 1968;38(1 Suppl):61–7. 10.1161/01.CIR.38.1S5.V-61 4894151

[76] Carasso S Mutlak D Lessick J: Symptoms in severe aortic stenosis are associated with decreased compensatory circumferential myocardial mechanics. *J Am Soc Echocardiogr.* 2015;28(2):218–25. 10.1016/j.echo.2014.09.006 25441330

[77] Attias D Macron L Dreyfus J: Relationship between longitudinal strain and symptomatic status in aortic stenosis. *J Am Soc Echocardiogr.* 2013;26(8):868–74. 10.1016/j.echo.2013.05.004 23768690

[78] Løgstrup BB Andersen HR Thuesen L: Left ventricular global systolic longitudinal deformation and prognosis 1 year after femoral and apical transcatheter aortic valve implantation. *J Am Soc Echocardiogr.* 2013;26(3):246–54. 10.1016/j.echo.2012.12.006 23306032

[79] Kamperidis V van Rosendael PJ Ng ACT: Impact of flow and left ventricular strain on outcome of patients with preserved left ventricular ejection fraction and low gradient severe aortic stenosis undergoing aortic valve replacement. *Am J Cardiol.* 2014;114(12):1875–81. 10.1016/j.amjcard.2014.09.030 25438916

[80] Dahou A Bartko PE Capoulade R: Usefulness of global left ventricular longitudinal strain for risk stratification in low ejection fraction, low-gradient aortic stenosis: results from the multicenter True or Pseudo-Severe Aortic Stenosis study. *Circ Cardiovasc Imaging.* 2015;8(3):e002117. 10.1161/CIRCIMAGING.114.002117 25681417

[81] Nagata Y Takeuchi M Wu VC: Prognostic value of LV deformation parameters using 2D and 3D speckle-tracking echocardiography in asymptomatic patients with severe aortic stenosis and preserved LV ejection fraction. *JACC Cardiovasc Imaging.* 2015;8(3):235–45. 10.1016/j.jcmg.2014.12.009 25682511

[82] Kusunose K Goodman A Parikh R: Incremental prognostic value of left ventricular global longitudinal strain in patients with aortic stenosis and preserved ejection fraction. *Circ Cardiovasc Imaging.* 2014;7(6):938–45. 10.1161/CIRCIMAGING.114.002041 25320287

[83] Di Salvo G Rea A Mormile A: Usefulness of bidimensional strain imaging for predicting outcome in asymptomatic patients aged ≤ 16 years with isolated moderate to severe aortic regurgitation. *Am J Cardiol.* 2012;110(7):1051–5. 10.1016/j.amjcard.2012.05.039 22728004

[84] Iida N Seo Y Ishizu T: Transmural compensation of myocardial deformation to preserve left ventricular ejection performance in chronic aortic regurgitation. *J Am Soc Echocardiogr.* 2012;25(6):620–8. 10.1016/j.echo.2012.02.005 22440541

[85] Olsen NT Sogaard P Larsson HB: Speckle-tracking echocardiography for predicting outcome in chronic aortic regurgitation during conservative management and after surgery. *JACC Cardiovasc Imaging.* 2011;4(3):223–30. 10.1016/j.jcmg.2010.11.016 21414568

[86] Onishi T Kawai H Tatsumi K: Preoperative systolic strain rate predicts postoperative left ventricular dysfunction in patients with chronic aortic regurgitation. *Circ Cardiovasc Imaging.* 2010;3(2):134–41. 10.1161/CIRCIMAGING.109.888354 20061517

[87] Park SH Yang YA Kim KY: Left Ventricular Strain as Predictor of Chronic Aortic Regurgitation. *J Cardiovasc Ultrasound.* 2015;23(2):78–85. 10.4250/jcu.2015.23.2.78 26140149 PMC4486182

[88] Nishimura RA Otto CM Bonow RO: 2014 AHA/ACC guideline for the management of patients with valvular heart disease: executive summary: a report of the American College of Cardiology/American Heart Association Task Force on Practice Guidelines. *J Am Coll Cardiol.* 2014;63(22):2438–88. 10.1016/j.jacc.2014.02.537 24603192

[89] de Isla LP de Agustin A Rodrigo JL: Chronic mitral regurgitation: a pilot study to assess preoperative left ventricular contractile function using speckle-tracking echocardiography. *J Am Soc Echocardiogr.* 2009;22(7):831–8. 10.1016/j.echo.2009.04.016 19505795

[90] Witkowski TG Thomas JD Debonnaire PJMR: Global longitudinal strain predicts left ventricular dysfunction after mitral valve repair. *Eur Heart J Cardiovasc Imaging.* 2013;14(1):69–76. 10.1093/ehjci/jes155 22848021

[91] Pandis D Sengupta PP Castillo JG: Assessment of longitudinal myocardial mechanics in patients with degenerative mitral valve regurgitation predicts postoperative worsening of left ventricular systolic function. *J Am Soc Echocardiogr.* 2014;27(6):627–38. 10.1016/j.echo.2014.02.008 24735653

[92] Smith LA Monaghan MJ: Monitoring of procedures: peri-interventional echo assessment for transcatheter aortic valve implantation. *Eur Heart J Cardiovasc Imaging.* 2013;14(9):840–50. 10.1093/ehjci/jet042 23580556

[93] Osman F Steeds R: Use of intra-cardiac ultrasound in the diagnosis of prosthetic valve malfunction. *Eur J Echocardiogr.* 2007;8(5):392–4. 10.1016/j.euje.2006.04.002 16762598

[94] Bartel T Bonaros N Edlinger M: Intracardiac echo and reduced radiocontrast requirements during TAVR. *JACC Cardiovasc Imaging.* 2014;7(3):319–20. 10.1016/j.jcmg.2013.07.010 24269262

[95] Corti R Biaggi P Gaemperli O: Integrated x-ray and echocardiography imaging for structural heart interventions. *EuroIntervention.* 2013;9(7):863–9. 24280159 10.4244/EIJV9I7A140

[96] Gao G Penney G Ma Y: Registration of 3D trans-esophageal echocardiography to X-ray fluoroscopy using image-based probe tracking. *Med Image Anal.* 2012;16(1):38–49. 10.1016/j.media.2011.05.003 21624845

[97] Kaiser M John M Heimann T: 2D/3D registration of TEE probe from two non-orthogonal C-arm directions. *Med Image Comput Comput Assist Interv.* 2014;17(Pt 1):283–90. 10.1007/978-3-319-10404-1_36 25333129

[98] Quaife RA Salcedo EE Carroll JD: Procedural guidance using advance imaging techniques for percutaneous edge-to-edge mitral valve repair. *Curr Cardiol Rep.* 2014;16(2):452. 10.1007/s11886-013-0452-5 24430014

