# Supplementary material for: Recent advances in echocardiography for valvular heart disease
Source: F1000Res. 2015 Sep 28;4(F1000 Faculty Rev):914. [Version 1] doi: 10.12688/f1000research.6446.1 (PMC4648195; doi:10.12688/f1000research.6446.1)
Supplement: Peer review history

# Peer review: Referee response for version 1

**DOI:** 10.5256/f1000research.6917.r10568

**Published:** 2015-09-28

**Authors:** Takahiro Shiota

**Affiliations:** 1 Cardiac Noninvasive Laboratory, Cedars-Sinai Heart Institute, Los Angeles, CA, USA

This review by Dr. Hahn from Columbia University covers major areas of the topic (recent advances in echocardiography in valvular heart disease) and is well written. With this, readers can appreciate her daily clinical experiences with new modalities such as three dimensional color Doppler echocardiography. In addition, recent major publications, including her own, are well chosen for the educational purpose.

I have read this submission. I believe that I have an appropriate level of expertise to confirm that it is of an acceptable scientific standard.

# Peer review: Referee response for version 1

**DOI:** 10.5256/f1000research.6917.r10567

**Published:** 2015-09-28

**Authors:** Jeroen J. Bax

**Affiliations:** 1 Department of Cardiology, Leiden University Medical Center, Leiden, Netherlands

I have read this submission. I believe that I have an appropriate level of expertise to confirm that it is of an acceptable scientific standard.

# Peer review: Referee response for version 1

**DOI:** 10.5256/f1000research.6917.r10566

**Published:** 2015-09-28

**Authors:** Judy Hung

**Affiliations:** 1 Massachusetts General Hospital, Boston, MA, USA

I have read this submission. I believe that I have an appropriate level of expertise to confirm that it is of an acceptable scientific standard.
